# Guilt, Shame and Compassionate Imagery in War: Traumatized German Soldiers with PTSD, a Pilot Study

**DOI:** 10.3390/jcm5100090

**Published:** 2016-10-20

**Authors:** Christina Alliger-Horn, Peter Lutz Zimmermann, Mervyn Schmucker

**Affiliations:** 1 Bundeswehrkrankenhaus Berlin, Abt. VIB Psychotraumazentrum, Scharnhorststr. 13, 10115 Berlin, Germany; peter1zimmermann@bundeswehr.org; 2 Klinik am Waldschlösschen, Zentrum für Psychotherapie, 01067 Dresden, Germany; irrt.de@gmail.com

**Keywords:** PTSD, combat, military, imagery rescripting, emotions, compassionate imagery, IRRT

## Abstract

Background: The consideration of specific trauma-associated emotions poses a challenge for the differential treatment planning in trauma therapy. Soldiers experiencing deployment-related posttraumatic stress disorder often struggle with emotions of guilt and shame as a central component of their PTSD. Objective: The purpose of this study was to examine the extent to which soldiers’ PTSD symptoms and their trauma-related guilt and shame may be affected as a function of their ability to develop compassionate imagery between their CURRENT SELF (today) and their TRAUMATIZED SELF (back then). Method: The sample comprised 24 male German soldiers diagnosed with PTSD who were examined on the Posttraumatic Diagnostic Scale (PDS) and two additional measures: the Emotional Distress Inventory (EIBE) and the Quality of Interaction between the CURRENT SELF and the TRAUMATIZED SELF (QUI-HD: Qualität der Interaktion zwischen HEUTIGEN ICH und DAMALIGEN ICH) at pre- and post-treatment and again at follow-up. The treatment used was imagery rescripting and reprocessing therapy (IRRT). Results: Eighteen of the 24 soldiers showed significant improvement in their PTSD symptoms at post-treatment and at follow-up (on their reliable change index). A significant change in trauma-associated guilt and shame emerged when compassionate imagery was developed towards one’s TRAUMATIZED SELF. The degree and intensity of the guilt and shame felt at the beginning of treatment and the degree of compassionate imagery developed toward the TRAUMATIZED SELF were predictors for change on the PDS scores. Conclusions: For soldiers suffering from specific war-related trauma involving PTSD, the use of self-nurturing, compassionate imagery that fosters reconciling with the traumatized part of the self can effectively diminish trauma-related symptoms, especially when guilt and shame are central emotions.

## 1. Introduction

In recent decades, clinical research in the field of psychotraumatology has made enormous advances. Today, many published studies are available pertaining to the efficacy of various trauma treatments [1], which has prompted scientific societies to begin recommending standards for the treatment of trauma patients (Arbeitsgemeinschaft der Wissenschaftlichen Medizinischen Fachgesellschaften (AWMF) [2]). Numerous studies and meta-analyses on the psychotherapeutic treatment of individuals suffering from posttraumatic stress disorder (PTSD) have examined the efficacy of empirically-established trauma therapies [1,3], as well as the effects of different treatment settings [4,5].

Schnyder et al. [6] described the central components that are, to a greater or lesser extent, part of all trauma-focused therapy approaches. Exposure-based, cognitive-behavioral therapies generally emphasize emotional processing of traumatic events with a particular focus on identifying and restructuring the cognitive processes that underlie and maintain the traumagenic emotions [7,8]. What most trauma-focused therapies implicitly have in common is the regulation and reorganization of central memory processes. A further common component of trauma-focused treatments involves psychoeducation, which includes the acquisition and the development of appropriate emotion-regulation strategies. The use of imaginal exposure (reliving) to facilitate the activation and processing of painful, trauma-related emotions is likewise a central component of exposure-based approaches.

While a number of trauma-focused treatments have received broad-based empirical support, relatively little is known about when, why and under what conditions empirically-supported treatments for PTSD are likely to be effective (or ineffective), and it remains unclear which specific interventions are best suited for which specific trauma types and characteristics [6,9]. Deciding which empirically-based treatment to employ in a clinical setting is made even more difficult by the fact that much of the trauma outcome research has focused on mono-symptomatic PTSD (without co-morbidity), which in turn has limited applicability to clinical practice [10]. As such, the process by which practicing clinicians can make informed decisions regarding what the best treatment fit may be for the specific trauma characteristics of a given patient remains unclear, and clinicians are left virtually on their own to choose the trauma treatment that they regard as the most appropriate for their trauma patients, which is essentially limited to the treatment(s) that they have been trained to implement [11].

If, as suggested by Benecke [12], mental disorders are regarded as manifestations of emotional dysregulation, then the activation and regulation of trauma-related emotions will necessarily play a crucial role in trauma-processing therapy. In this context, prominent trauma-related emotions are not independent of the type and nature of the traumatic event experienced by the patient, which would appear to be particularly relevant to soldiers. Verstrael et al. [13] discussed the relatively limited effects that even empirically well-established exposure-based, trauma-processing treatments (e.g., prolonged exposure, eye movement desensitization) have on soldiers and draw attention to the fact that the specific trauma-related emotions that emerge following deployment have not been sufficiently examined. In addition to fear and helplessness, e.g., following direct combat action, attacks and battle injury during deployment, intense emotions of guilt and shame frequently accompany war experiences [14,15,16]. Such psychic trauma often leads to a deeply shaken self-image and worldview, together with a questioning of personal moral beliefs, which are at the core of solders’ traumatic war experiences [15,16]. As with other soldiers throughout the world, German soldiers are confronted with highly disturbing events related to human suffering often associated with guilt and shame. In a sample of German soldiers conducted by Wittchen et al. [17], such events may include firing at the enemy (18%) and being confronted with destruction and violence in the country of deployment (70%).

A primary objective of exposure-based, trauma-focused therapies has been to activate and process the specific, central trauma-related emotions of patients and to challenge the underlying cognitions and beliefs that maintain them. In recent years, imagery rescripting approaches have been increasingly employed to treat overarching negative emotions, such as guilt and shame [9,18,19,20,21]. The use of imagery-based, trauma-focused interventions that emphasize the development of mastery and self-nurturing imagery has shown promising signs in the treatment of deployment-related traumata in soldiers, especially in helping them to cope more effectively with their trauma-related emotions. In particular, the development of self-nurturance and self-care imagery that facilitates a more compassionate and conciliatory view towards the wounded, injured parts of the self appears to be especially useful with soldiers [11].

The purpose of this pilot study, which draws on a sample of 24 war-traumatized male soldiers with PTSD, is three-fold: (1) to examine the effect that guilt and shame have on changes in trauma symptomatology through an exposure-based, imagery-focused trauma treatment: imagery rescripting and reprocessing therapy [21]; (2) to examine whether the development of mastery and self-compassionate imagery created in the IRRT sessions has an impact on trauma symptoms; and (3) to identify and describe any changes in the range of emotions that emerge as part of the imagery sessions.

### Imagery Rescripting and Reprocessing Therapy

IRRT is an imagery-based, trauma-focused treatment that involves activating, confronting and modifying traumatic images and related maladaptive attributions, beliefs and schemas, which are processed both visually and verbally during high states of affective arousal within the context of a Socratic-facilitated intrapersonal dialogue between the patient’s CURRENT SELF (today) and TRAUMATIZED SELF (back then). The primary goal of IRRT is to provide patients with a mental structural framework within which to activate, modify and emotionally process distressing traumatic imagery and to use compassionate imagery as a means of enhancing one’s ability to self-calm, self-nurture and emotionally self-regulate. IRRT comprises three distinct phases: (1) imaginal exposure: visual and affective reliving of the entire traumatic scene; (2) mastery imagery: visualizing one’s CURRENT SELF entering the trauma scene to confront and disempower the perpetrator; and (3) self-nurturing/self-compassionate imagery: visualizing one’s CURRENT SELF nurturing, calming, understanding and reassuring the TRAUMATIZED SELF, which often involves the active processing and re-processing of an array of non-fear emotions (e.g., guilt, shame, anger, powerlessness, abandonment, betrayal).

## 2. Method

### 2.1. Study Participants and Intervention

Inclusion criteria for this study were: (a) PTSD as a primary diagnosis (in accordance with the International Classification of Diseases (ICD-10) and DSM-IV)) ensuing from traumatic events occurring during Bundeswehr missions abroad; (b) voluntary participation in the study involving eight weeks of inpatient treatment at the Bundeswehr Hospital Berlin; and (c) experiencing the traumatic event no less than 6 months and no more than 16 years before treatment.

Exclusion criteria were: (a) other types of trauma not associated with a Bundeswehr mission abroad (e.g., child sexual trauma); (b) complex Type II trauma (especially non-military traumata); (c) acute suicidal ideation; (d) acute psychotic disorders; (e); severe physical disorders; and (f) substance addiction.

All study participants underwent treatment during their inpatient stay at the Bundeswehr Hospital Berlin and were examined directly upon admission with the psychometric instruments noted in the next section. Participants were admitted via the outpatient Department of Psychiatry at the Bundeswehr Hospital Berlin, where a medical specialist for psychiatry and psychotherapy had previously determined, based on an initial clinical assessment, that inpatient trauma treatment was indicated.

Clinical diagnoses were established during the first inpatient week, which was then followed by a 3–4 week stabilization phase that included teaching the participants progressive muscle relaxation and safe-place imagery. In addition, all 24 participants received three 50-min individual sessions with their therapists. In accordance with regular treatment intervals, the patients were then temporarily discharged for 4–5 weeks, after which they were re-admitted as inpatients to the hospital.

During their second inpatient stay, patients received three weekly individual sessions of IRRT trauma treatment over a period of six weeks, with sessions lasting from 50 min to 100 min each. Therapists received a 90-min supervision session each week (from the senior author). The standard 3-phase IRRT treatment protocol (a modification in the standard 3-phase IRRT treatment protocol was made with patients when no identifiable perpetrator was present, which involved applying IRRT Phases 1 and 3 only) was implemented in accordance with Schmucker and Köster [21]. Special therapeutic emphasis was on the 3rd phase, which involved patients activating and processing deep-seated, negative internal representations of the self associated with their war traumas, with a particular focus on targeting and modifying their maladaptive beliefs relating to guilt and shame. A primary goal of these 3rd phase imaginal interactions between the different parts of the self was for the CURRENT SELF to develop compassionate imagery towards the TRAUMATIZED SELF as a means of enhancing self-nurturance and developing a more conciliatory and empathic attitude towards oneself [11].

During their 6-week hospital stay, all 24 patients also participated in the general treatment program offered by the psychiatric ward, which included occupational therapy, sports and exercise therapy and massages. There were no patient dropouts.

### 2.2. Measuring Instruments

The clinical diagnoses were established with the SCID I and II instruments (Structured Clinical Interview for DSM-IV, Axis II: Wittchen et al. [22]). The PDS (German translation of the Posttraumatic Diagnostic Scale by Ehlers et al. [23], a self-rating, diagnostic instrument designed to assess PTSD in accordance with DSM-IV and ICD-10 criteria, was administered at pre-treatment, post-treatment and at 3-months’ follow-up. The internal consistency for the total score of the German version of the PDS has been elsewhere reported to be 0.94 [24].

Prior to therapy, patients completed a self-report questionnaire assessing their current feelings about the most distressing traumatic experiences they had had during deployment. This questionnaire (Emotional Distress Inventory-Soldier-Version (EIBE-Soldier-Version)): Schmucker et al. [25]; see Appendix A.1) was adapted for use with military samples from the original EIBE version developed by Schmucker and Köster [21]. Patients were instructed to assess the intensity of their current emotions on a Likert scale of 0–5. Immediately following the first and last IRRT sessions and at 3-month follow-up, patients received an additional questionnaire (Quality of Interaction (QUI)-HD: translated form the Germen version: Qualität der Interaktion zwischen HEUTIGEN ICH UND DAMALIGEN ICH (QUI-HD Soldatenversion): Alliger-Horn et al. [26]; see Appendix A.2). The QUI-HD questionnaire was designed to analyze the quality of interaction between the CURRENT SELF and the TRAUMATIZED SELF. Participants were asked to assess their present feelings towards their TRAUMATIZED SELF. Two additional imagery-related questions were included in later data analyses: (1) How much emotional distance did you experience in the imagery session today between your CURRENT SELF and your TRAUMATIZED SELF? (2) How difficult was it for you to be supportive to your TRAUMATIZED SELF in the imagery session today?

As all questionnaires are used in regular clinical diagnoses and therapeutic treatment planning, no approval was required from the ethics committee. All participating patients had given their consent to the examination.

### 2.3. Data Analysis

In a first step, the PDS sample means and standard deviations were calculated and analyzed for statistical differences using χ^2^ and *t* tests. Given the available dataset, a dropout and missing data analysis was not necessary. An analysis of variance with repeated measurements was conducted to determine the degree to which trauma severity overall had changed on the PDS over time. Effect sizes were estimated using Cohen’s standardized mean difference *d* [27]. The effect size for the PDS was calculated at post-treatment and at 3-months’ follow-up. A reliable change index (RCI) was calculated in accordance with Jacobsen and Truax [28]. Significant improvement in clinical symptoms was assumed for an RCI of ≤−1.96 and for *p* ≤ 0.05. In order to determine clinical significance, the number of PTSD-diagnosed patients and the PTSD symptom severity on the PDS were also analyzed in accordance with Foa et al.’s [29] recommendations: moderate, a PDS total score of 11–20; moderate to severe, a PDS total score of 21–35; severe, a PDS total score >35.

In the second part of the study, the EIBE questionnaire (Soldier Version) was used at pre-treatment to assess guilt and shame associated with the most distressing traumatic event witnessed during deployment. The change in the perceived emotional distance between the CURRENT SELF and the TRAUMATIZED SELF and the change in perceived emotional support offered by the CURRENT SELF to the TRAUMATIZED SELF (QUI-HD) were analyzed as predictors of therapeutic changes in trauma symptoms on the PDS total score at three different points of measurement (t1, t2, t3) by means of regression analyses. Changes in the visualized distance between the TRAUMATIZED SELF and the CURRENT SELF, as well as changes in the visualized emotional support offered by the CURRENT SELF to the TRAUMATIZED SELF were calculated as the difference between t1 and t2 and between t1 and t3. The difference in the PDS total score was used as the dependent variable.

The third part of the study comprised the statistical analyses of the reported change in emotions vis-à-vis the CURRENT SELF and the TRAUMATIZED SELF at pre-treatment (t1), at post-treatment (t2) and at 3-months’ follow-up (t3). For this purpose, mean values were compared.

## 3. Results

The 24 male patients participating in this study were all diagnosed with PTSD, as assessed on the SCID I. A majority of these patients (67%) also had co-morbid diagnoses, which included anxiety disorders (41.2%), depressive disorders (12.3%) and Axis II personality disorders (42.7%), of whom 31.2% presented with Cluster C disorders. The average age of the patients at the time of the initial examination was *M* = 39.3 years (SD = 9.0, range = 27–53 years). At the time of their hospitalization, all patients had been deployed in one or more missions abroad (Kosovo and Afghanistan) for an average total period of *M* = 14.6 months (SD = 15.5, range = 3–77 months in action), and all had experienced specific war-related traumata, which included participation in active combat, the killing of adversaries, witnessing atrocities against civilian populations, the killing of women and children, the opening of mass graves, the direct wounding of fellow soldiers in combat and the killing of adversaries at close range.

The pre-treatment total PDS score was *M* = 32.25 (SD = 8.67, range 14–45), which lies in the moderate to severe PTSD symptom range. At post-treatment, there was a significant change in total PDS scores (*M* = 25.88, SD = 8.57; χ^2^ (1, *N* = 23) = 4.89, *p* ≤ 0.001). At the three-month follow-up, the significant positive changes in clinical symptoms were maintained (*M* = 21.83, SD = 10.44; χ^2^ (1, *N* = 23) = 4.99, *p* ≤ 0.001). The ANOVAs also revealed significant positive changes in the PDS total score (*F*(1,23) = 24, *p* ≤ 0.001; η^2^ = 0.510) at all three measurement points. The post-treatment effect size was *d* = 0.98, while the effect size at three-months’ follow-up was *d* = 0.99. The reliable change index (RCI) based on the post-treatment PDS scores revealed that 71% of the patients (*n* = 17) had shown a significant improvement in their PTSD symptoms (RCI ≤ −1.96; *p* ≤ 0.05); for 21% (*n* = 5), the clinical symptoms had remained unchanged; and for 8% (*n* = 2), the symptoms had worsened (RCI ≥ 1.96; *p* ≤ 0.05). Similarly, the reliable change index based on the three-month follow-up PDS scores indicated that for 75% (*n* = 18) of patients, the clinical symptoms had significantly improved (RCI ≤ −1.96; *p* ≤ 0.05); for 17% (*n* = 4), the symptoms had remained unchanged; and for 8% (*n* = 2), the symptoms had worsened (RCI ≥ 1.96; *p* ≤ 0.05). This worsening of symptoms appeared to be caused by crises experienced during the course of treatment (e.g., loss of partnership, denial of compensation by the Bundeswehr).

As expected, the regression analysis (see Table 1) revealed a significant effect of the experienced emotional distance between the CURRENT SELF and the TRAUMATIZED SELF. Specifically, trauma symptoms were more likely to improve in patients who perceived relatively less emotional distance between the CURRENT SELF and the TRAUMATIZED SELF at post-treatment and who demonstrated an enhanced ability to offer emotional support to the TRAUMATIZED SELF. The emotions of guilt and shame reported at pre-treatment were likewise found to be predictors of changes in trauma symptoms. Patients who had reported particularly strong feelings of shame and guilt at pre-treatment demonstrated more pronounced changes in their PDS total scores at the end of treatment. A similar picture emerged at follow-up. An improved ability to offer emotional support to one’s TRAUMATIZED SELF and a reduced emotional distance between one’s CURRENT SELF and TRAUMATIZED SELF also had a significant long-term effect on the positive changes noted in the PDS scores. The initially reported shame and guilt that patients felt when recalling the traumatic event were predictors of long-term changes in PDS scores as a result of the imagery treatment interventions.

Table 2 shows the emotional changes between the CURRENT SELF and TRAUMATIZED SELF at the three measurement points. At post-treatment, significant positive changes were observed in nearly all of the emotions vis-à-vis the TRAUMATIZED SELF. The only reported emotions that had not significantly changed were “love” and “care”. The follow-up data (t3) revealed that these results were largely robust. At each of the measurement points (t2, t3), reported feelings of shame, guilt and helplessness were found to have decreased significantly.

## 4. Discussion

During combat missions, soldiers are exposed to a range of traumatic events, some of which are not necessarily associated with fear and helplessness, but which may trigger a profound sense of moral injury [16,30]. In a military context, the killing of people is a notable predictor of PTSD and depression, as well as of suicidal ideation [14] and is associated with specific emotions. Frequently, guilt and shame are the central trauma-related emotions reported by soldiers following combat missions. In their study, Nazarov et al. [31] noted a connection between moral traumas, deployment-related psychological disorders and symptoms of guilt and shame with soldiers. Similarly, the findings reported by Hellenthal et al. [32] underscored a direct link between posttraumatic symptoms and “moral injury” experienced by German soldiers deployed in Afghanistan.

The purpose of this pilot study was to examine the extent to which changes in specific trauma-related emotions like guilt and shame, experienced by soldiers with PTSD ensuing from war trauma, can affect therapeutic change in trauma symptoms. Also examined in this study was the role of compassionate imagery involving self-nurturance and self-care vis-à-vis the injured and traumatized parts of the self. This is based on the assumption that the expression of compassion is a basic, inborn human ability that can be weakened by traumatic experiences (e.g., war) or strengthened through focused training. The use of compassionate imagery as a means of enhancing one’s ability to self-calm, self-nurture and emotionally self-regulate is a central component of IRRT. In this context, compassionate imagery is considered to be a special kind of “mastery imagery” in the treatment of traumatized soldiers in this pilot study.

In recent years, imagery rescripting approaches have been successfully used to treat depression and a range of anxiety disorders, as well as nightmares, OCD, bulimia nervosa and personality disorders [18,33,34,35,36,37]. Imagery interventions have also become a key element of empirically-demonstrated exposure-based approaches in the treatment of PTSD and other trauma-related symptoms [6,8,19,20,38]. In a study conducted with victims of industrial injury suffering from PTSD, Grunert et al. [9] demonstrated the effective use of IRRT (in contrast to extinction-based exposure therapy) in treating trauma-related guilt. The first documented use of IRRT in the treatment of German active-duty soldiers suffering from war trauma was reported in a recent study by Alliger-Horn et al. [11]. In particular, the focus on developing self-nurturing and self-conciliatory imagery with the TRAUMATIZED SELF in Phase 3 of IRRT appeared to play a decisive role in fostering significant positive therapeutic change. Results of this pilot study represent a promising first step in: (1) the use of imagery procedures as a means of producing changes in the trauma symptoms of soldiers experiencing intense feelings of guilt and shame; and (2) affecting long-term positive changes in trauma symptomology relating to the emotional quality of imagery interactions (verbal and non-verbal) between the CURRENT SELF and the TRAUMATIZED SELF (including positive changes in the way one views the TRAUMATIZED SELF).

These findings are in accordance with previously-published studies on the use of imagery rescripting for non-fear emotions [19,39] and builds on the results reported by Alliger-Horn et al. [11] in which IRRT was successfully employed with German soldiers. The use of imagery focused procedures has also been described in more recent training programs focusing on soldiers from other countries with PTSD resulting from war-related traumata [40]. As this is an initial non-randomized pilot study, direct causation cannot be inferred. Nonetheless, these are encouraging results for the use of imagery interventions, especially the use of compassionate imagery, in exposure-based, trauma therapy focused on specific war-related traumata. The emotional “reconciliation” of the two parts of the self, activated through a visual dialogue between the CURRENT SELF and the TRAUMATIZED SELF (in the third phase of IRRT), appears to have a particularly positive effect on trauma-related symptoms of guilt and shame stemming from deployment (e.g., killing of adversaries, recognition of one's own transgressions and those of others). The improved ability of soldiers to provide themselves with emotional support via imagery techniques (e.g., compassionate imagery) not only appears to foster positive changes in their trauma symptoms, but may play a central role in their overall recovery and general well-being, as well.

## 5. Limitations

Because of the small sample size, the results of this pilot study prohibit us from making generalized and causal statements or from drawing conclusions about the treatment of war-related guilt and shame with imagery interventions. Methodological limitations include the lack of randomization of patient selection, lack of a control group, limited usage of psychometric measures (including measures related to the DSM 4) and not including co-morbidity in the data analyses. In addition, the sample comprised male traumatized soldiers only, which limits the applicability of these findings to other trauma groups. In spite of these limitations, the results of this study may be viewed as an early indication of a promising treatment.

## 6. Conclusions

The therapeutic treatment of deployment-related disorders and especially of PTSD poses a challenge for healthcare systems. Psychotherapy and especially exposure-based trauma-focused approaches foster and promote emotion-regulation strategies with patients [12]. This suggests that exploring the significance of specific trauma-related emotions is a key component of trauma-focused therapies. For patients with an active military background, the type of trauma (e.g., fear-based vs. guilt- and shame-based) appears to play a significant role in the development of various deployment-related disorders. For patients suffering from the after-effects of specific war-related traumas, it appears particularly useful to enhance one’s ability to reconcile with the TRAUMATIZED SELF by promoting self-nurturance, self-care and emotional self-regulation via compassionate imagery. This applies especially when guilt and shame are central trauma-related emotions.

In summary, identifying and labelling the specific emotions and idiosyncratic beliefs closely associated with a traumatic event could further help clinicians to make better, more informed decisions regarding what specific, trauma-focused interventions may be the best treatment fit for the specific trauma characteristics of a given patient. Targeted imagery rescripting interventions appear to be a promising element for the treatment of specific traumata and one that deserves further examination. Further studies that compare the various imagery methods in the treatment of guilt and shame, especially the use of compassionate imagery, could enhance our understanding of the underlying mechanisms at work in the treatment and recovery from trauma-related disorders and further enable therapists to respond more effectively to the special needs of specific traumatized subgroups.

## Figures and Tables

**Table 1:** Hierarchical multiple regression analyses (differences at t1-t2 and t1-t3 with PDS total score as dependent variables).

	Variable	Regression Coefficient B	Standardised Coefficient Beta	Standard Error SE	Significance *p*
Short-term (*N* = 24)	**PDS**				
	Guilt	1.368	0.455	0.424	0.004
	Shame	1.307	0.381	0.419	0.006
	Diff_support	2.231	0.577	0.535	0.001
	Diff_distance	−1.873	−0.531	0.447	0.000
Long-term (*N* = 24)	**PDS**				
	Guilt	3.494	0.494	0.985	0.002
	Shame	0.391	0.080	0.682	0.574
	Diff_support	2.836	0.448	0.900	0.005
	Diff_distance	−2.471	−0.428	0.828	0.008

Note: *R*^2^ (t1-t2)_PDS_ = 0.736; *R*^2^ (t1-t3)_PDS_ = 0.648.

**Table 2:** Change in emotions between CURRENT SELF and TRAUMATIZED SELF (t1-t2 and t1-t3).

Attitude of the CURRENT SELF about the TRAUMATIZED SELF	t1-t2 t1-t3 *N* = 24 M (SD)	*t*-Test (t1-t2) *t*-Test (t1-t3) *N* = 24 M (SD)
Anger and Rage	0.667 (1.090) 0.458 (1.285)	2.996 ** 1.748 n.s.
Helplessness	2.208 (1.668) 2.042 (1.732)	6.488 *** 5.776 ***
Care	−0.667 (1.579) −0.375 (1.837)	−2.069 n.s. −1.000 n.s.
Sadness	1.458 (1.719) 1.125 (1.702)	4.156 *** 3.238 **
Love/Affection	−0.625 (1.861) −0.167 (2.180)	−1.646 n.s. −0.374 n.s.
Rejection/Aversion	0.958 (1.732) 0.708 (1.546)	2.711 * 2.245 *
Guilt	0.792 (1.382) 0.708 (1.459)	2.805 * 2.378 *
Shame	1.292 (1.459) 1.167 (1.494)	4.337 *** 3.826 ***

Note: *t*-test, α = 0.05., *p* ≤ 0.01 *, *p* ≤ 0.005 **, *p* ≤ 0.001 ***.

## Appendix A

### Appendix A.1. EIBE–Soldier-Version (Emotional Distress Inventory) [25]

Name:_________________________________ Date:________________________					
What incident during your mission abroad would you like to work on today in the IRRT session? ___________________________________________________________________________					
How much time has passed since this incident? ____________________________________					
What emotion(s) do you associate with this incident when you think about it *today*?					
*Anger*					
0 not at all	1	2	3	4	5 very strong
*Helplessness*					
0 not at all	1	2	3	4	5 very strong
*Sadness*					
0 not at all	1	2	3	4	5 very strong
*Emotional numbing*					
0 not at all	1	2	3	4	5 very strong
*Guilt*					
0 not at all	1	2	3	4	5 very strong
*Shame*					
0 not at all	1	2	3	4	5 very strong
*Fear*					
0 not at all	1	2	3	4	5 very strong
*Disgust*					
0 not at all	1	2	3	4	5 very strong
*Horror*					
0 not at all	1	2	3	4	5 very strong
*Sense of unreality*					
0 not at all	1	2	3	4	5 very strong

### Appendix A.2. QUI-HD (Quality of interaction between the CURRENT SELF and the TRAUMATIZED SELF) [26]

Name:___________________________________ Date:_______________________					
How much emotional distance did you experience in the imagery session today between your current self and your traumatized self?					
0 not at all	1	2	3	4	5 very distant
How difficult was it for you to be supportive of your past self in this imagery session?					
0 not at all	1	2	3	4	5 very difficult
What were your feelings about your past self on deployment in this imagery session?					
*Anger/Rage*					
0 not at all	1	2	3	4	5 very strong
*Helplessness*					
0 not at all	1	2	3	4	5 very strong
*Care*					
0 not at all	1	2	3	4	5 very strong
*Sadness*					
0not at all	1	2	3	4	5very strong
*Love/ Affection*					
0 not at all	1	2	3	4	5 very strong
*Rejection/ Aversion*					
0 not at all	1	2	3	4	5 very strong
*Guilt*					
0 not at all	1	2	3	4	5 very strong
*Shame*					
0 not at all	1	2	3	4	5 very strong
